# Anxiety and depression among Tunisian women victims of domestic violence

**DOI:** 10.1192/j.eurpsy.2022.810

**Published:** 2022-09-01

**Authors:** R. Jbir, L. Aribi, W. Abid, I. Jbir, F. Charfeddine, S. Ellouze, J. Aloulou

**Affiliations:** 1 Hedi chaker, Hospital, Sfax, Tunisia; 2 Hedi Chaker University Hospital, Psychiatry B, Sfax, Tunisia

**Keywords:** women, Depression, Anxiety, domestic-violence

## Abstract

**Introduction:**

Violence against women is now widely recognized as an important public health problem with substantial consequences on mental health, that is why health professionals should be identifying, preventing, and responding to violence against women more effectively.

**Objectives:**

To study the prevalence and predictors of anxiety and depression among women victim of domestic violence

**Methods:**

Our study was descriptive and analytical cross-sectional, carried out with women examined in the context of medical expertise, from May until October 2021. An anonymous survey was asked to these ladies. The HADS was used to screen for anxiety and depression

**Results:**

75 responses was collected The age group of 26-35 years represents the highest percentage 44%. 98.7%were victim of verbal violence,94.7% of physical violence, 97.3% of psychological violence and 54.7 % of sexual violence According to the HAD, anxiety was retained in 72% and depression in 56%. Women who filed for divorce developed more depression (p=0.01). Women with a history of infertility were more anxious than others (p=0.025). Anxiety and depression were significately correlated with : the husband alcohol (p=0.01) and cannabis consumption(p=0.015). The ladies victims of sexual violence such as an unusual type of relationship developed more anxiety (p=0.045). An history of aggression during pregnancy was a risk factor of anxiety (p=0.035)

**Conclusions:**

Our work has shown the association between violence against women and anxiety-depressive symptoms. The results of our work inspire us to reflect on and develop actions on the scourge of violence against women in a conjugal environment and its psychological repercussions.

**Disclosure:**

No significant relationships.

